# Telemedicine Assessment for the Mental Health of Rural Residents Based on the Safety Degree of Housing in Seismically Active Regions

**DOI:** 10.3389/fpubh.2021.604298

**Published:** 2021-08-02

**Authors:** Yin Pan, Ruihang Xie, Qin Yan, Tiejun Zhou

**Affiliations:** ^1^College of Architecture and Urban Planning, Chongqing Jiaotong University, Chongqing, China; ^2^School of Architecture and Urban Planning, Chongqing University, Chongqing, China; ^3^School of Smart Urban Design, Chongqing Jianzhu College, Chongqing, China

**Keywords:** telemedicine assessment, mental health, housing safety, Kessler 6, seismically active regions, housing structural forms

## Abstract

Earthquakes inevitably affect the mental health of local residents. In seismically active regions of Southwest China, local rural residents' dilapidated housing with poor seismic performance aggravates the impacts of earthquakes on their mental health. These residents' mental health is difficult to recognize because of the lack of appropriate assessment methods. In addition, rural residents in the area have a low socioeconomic status and cannot access adequate mental treatment. Thus, telemedicine could be an effective approach to assist mental health practice in such areas. However, the lack of telemedicine assessment factors in these areas makes it difficult to complete the correct triage and prioritization of rural residents' mental health quickly and effectively. To provide a foundation for applying telemedicine to assess the risk of mental health problems that rural residents in seismically active regions experience, this paper studied whether the degree of safety of housing can affect mental health. In this study, nine villages near the epicenter of the 2019 6.0-magnitude earthquake in Changning County, China were randomly selected, and 162 valid questionnaires were completed. SPSS statistical software was used to analyze the collected data. First, the satisfaction of rural residents with the degree of safety of housing significantly affected the K6 score and whether they suffered from mental problems. Second, the mental health of rural residents living in reinforced concrete frame structure housing was obviously superior to that of those living in other types of housing. Next, the most significant factor affecting mental health was the degree of wall cracks. Finally, a new approach was developed to assess and prioritize the mental health of rural residents by using degrees of housing safety and smart technology in seismically active regions. The telemedicine assessment approach is expected to be used in the future for mental health evaluation and the large-scale data scoring of rural residents.

## Introduction

Earthquakes are one of the most destructive and frequent natural disasters in human history. Given their destructiveness and unpredictability, they have caused many human deaths and injuries, inevitably disturbing the mental health of local residents. The mental health problems caused by earthquakes has received considerable attention worldwide. In recent years, many countries, such as Japan (1), Italy (2), New Zealand (3), and China (4), have reported that many local residents experienced mental health problems in the aftermath of an earthquake. In China, Sichuan Province is considered a seismically active region. The province is located in Southwest China, bordering the Qinghai–Xizang Plateau. Frequent earthquakes occur due to the Longmenshan fault zone. Furthermore, many major earthquakes, such as the Wenchuan 8.0 earthquake in 2008, the Lushan 7.0 earthquake in 2013 and the 2019 6.0-magnitude earthquake in Changning County, have severely affected the world. Earthquakes have become the most destructive and frequent natural disaster in this region. In particular, after the Wenchuan earthquake in 2008, the devastating environmental damage and economic losses, coupled with the terrible casualty rate, made the mental problems of the local residents in the area more apparent. A previous study found that 1 month after the Wenchuan earthquake, the initial prevalence of posttraumatic stress disorder (PTSD) among 409 survivors in Qingchuan County was estimated to be as high as 62.8% (5).

Almost all earthquake injuries are directly or indirectly caused by building collapses or falling objects. Therefore, in the areas in which earthquakes occur frequently, under the influence of earthquakes, mental health has a natural relationship with building safety. For example, existing studies have found that after an earthquake, the greater the damage to residents' houses is, the more likely the residents are to suffer from mental problems (6, 7). Given that Sichuan Province is in an earthquake fault zone, especially after the Wenchuan earthquake, all buildings in Sichuan Province have been required to implement earthquake resistance. In particular, building safety in cities is relatively high. Even in most rural areas, under the strong promotion of the Chinese government, some dilapidated houses were demolished or renovated in conjunction with shantytown renovations. However, these policies do not include all buildings, nor can they prevent all earthquake casualties. In the 2019 Changning earthquake, 13 people were still killed, and 226 were injured. Therefore, compared with urban areas, rural areas face a greater risk of casualties during earthquakes.

In fact, we conducted a presurvey in the rural areas of Changning County after the 2019 Changning earthquake and found that local rural residents believed that the extraction of shale gas in the area had directly resulted in frequent earthquakes, the earthquake trend would persist if the extraction continued, and they would not be anxious if their houses were safe. Furthermore, we also found that most local residents were old people who had very limited capacity to obtain earthquake information on the Internet and usually obtained earthquake information from relatives and the government. Therefore, the degree of housing safety could be considered an extremely important factor for these rural residents, and whether the degree of housing safety can affect their mental health deserves further exploration and study.

At present, considerable research has been conducted on the mental health of residents after an earthquake. For example, many studies have found that after an earthquake, residents may experience mental health problems such as acute stress disorder, PTSD, anxiety, and depression (2, 8, 9). In addition, the common risk factors cited for postearthquake mental problems, including gender, age, educational background, social support, earthquake exposure, previous mental state, and severity of housing and property damage, have also received considerable attention (1, 4, 6, 10). Postearthquake experiences (such as homelessness and temporary housing types) have also been reported to be related to the severity and long-term effect of trauma (11). Thus, the effect of earthquake disasters on mental health and relevant measures have been an important research topic in the disaster literature in epidemiology, psychology, and social science.

Many studies have been conducted on the intervention methods of the mental health of residents in the aftermath of an earthquake. First, the treatment plans from the medical aspect include not only conventional drug treatments but also psychological therapy (12). Some interventions based on culture, such as Chinese calligraphic handwriting and acupuncture, have also had positive effects (13, 14). Finally, governmental and non-governmental programs that provide social and economic support, such as sustainable collaborative communities, were suggested to improve the living standards of residents in previous studies (15–17).

Indeed, the Chinese government has acknowledged rural residents' mental health in seismically active regions and has been seeking to improve the safety and health of rural residents in the region through relocation and urbanization measures. Therefore, the “Beautiful Countryside,” “New Countryside Construction,” and “New Urbanization” policies have been strongly supported and encouraged by the government in these areas. However, many rural residents continue to live in their original houses. Their houses may have some safety problems, but these problems are insufficient to urge the government to subsidize their repair and renovation. In addition, the lack of mental health professionals such as psychiatrists, clinical psychologists and psychiatric nurses and limited access to and distribution of these services are among the several obstacles in improving rural residents' mental health in seismically active regions of Southwest China. Rural residents also have a low socioeconomic status and cannot afford adequate mental treatment in cities. These reasons have made mental health interventions unavailable and expensive for these people.

Fortunately, telemedicine can be an effective method to assist healthcare. Such interventions have known to be feasible and applicable in earthquake settings and contexts. Two reviews stated that the appropriate use of telemedicine is crucial for risk management and emergency nursing practice during earthquakes (18, 19). Existing studies reported that telemedicine was used to assist plastic surgery, paraplegics, cardiopulmonary medicine, intensive care and other medical treatments during earthquake relief (20–24). Despite some authors developed telemedicine systems to support treatments and interventions (25–27), studies on the use of telemedicine for mental health in earthquake settings are limited.

In fact, telemedicine can provide mental assessment and treatment to rural and isolated areas that are inaccessible and out of reach by mental health professionals (28). The potential of telemedicine to improve the quality and extent of mental health services for rural, remote, and isolated populations has been demonstrated (29). Augusterfer et al. stated that telemental health was applied in distant education, guidance, management and case consultations to respond Haiti earthquake (30). Another study conducted by Qadir et al. focused on an online treatment in Pakistan, where some local residents were trained as professional workers to diagnose if residents have occurred mental problems, such as PTSD and anxiety (31). In addition, Ruzek et al. noted that a mobile app named PTSD coach for providing psychoeducation were downloaded and used widely after disasters and wars (32). But these researchers did not provide an appropriate method to complete the correct triage and prioritization of mental health, which may result in scarce resources cannot be used correctly, effective treatment priorities are neglected, and mental treatments delayed.

We envision that telemedicine assessment using housing safety degree can be applied to achieve correct and appropriate triage and treatment after earthquakes for two reasons. First, Southwest China that is mainly mountainous and sparsely populated has a vast area and complex landforms. For example, the area of Sichuan Province is larger than that of Germany and Japan. Meanwhile, the low per capita GDP in Southwest China means that local rural residents are unwilling to pay for the transportation to urban hospitals to diagnose mental problems. And nurses and psychologists are not physically available to help rural residents with mental problems. In telemedicine, these residents can be quickly and conveniently prioritized for treatment by assessing their degree of housing safety, which is easy to extract and judge, thereby obtaining a quick overview of the overall situation. Second, for rural residents, discussing mental health with others is a humiliating and awkward behavior (33). Consequently, certain telemedicine assessments and prioritization based on degrees of housing safety can avoid the awkwardness and resistance of rural residents. However, how the degree of housing safety affects the mental health of rural residents in seismically active regions is still unclear at present, which leads to the lack of telemedicine assessment factors in this area, and it is difficult to assess and prioritize the mental health of rural residents quickly and effectively.

Therefore, the main aim of this study is to explore, through interviews and surveys, whether the degree of housing safety in seismically active regions can affect the mental health of rural residents. Another aim is to explore the relationship between housing safety and the mental health of rural residents. The study will help us to evaluate determinants of the degree of housing safety and find potential protection factors to provide suggestions for future housing design and repair interventions and provide a foundation for developing a new telemedicine approach to assess and prioritize rural residents' mental health.

The remainder of this paper is organized as follows. The next section presents a summary of the study site, data collection, questionnaire design and data analysis. Section Results presents the research results achieved through statistical analysis. Section Discussion elaborates on the factors of the degree of housing safety affecting the mental health of rural residents and a new approach for telemedicine assessment. The last section concludes with contributions and future research.

## Methodology

### Case Study Site and Data Collection

**Figure 1:**
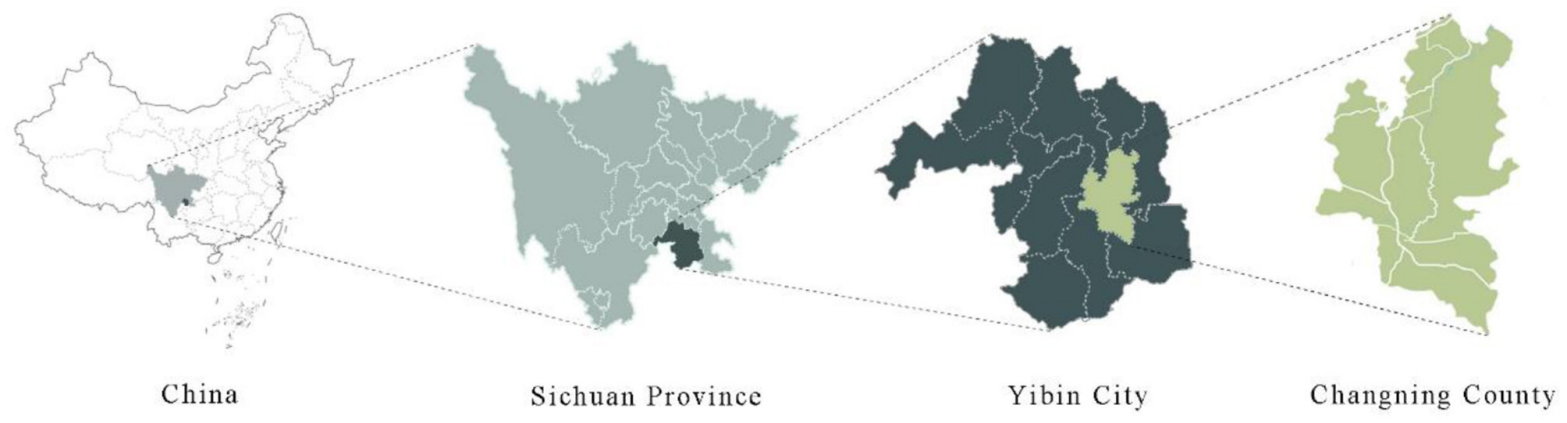
Location of the study site: Changning County, China.

**Figure 2:**
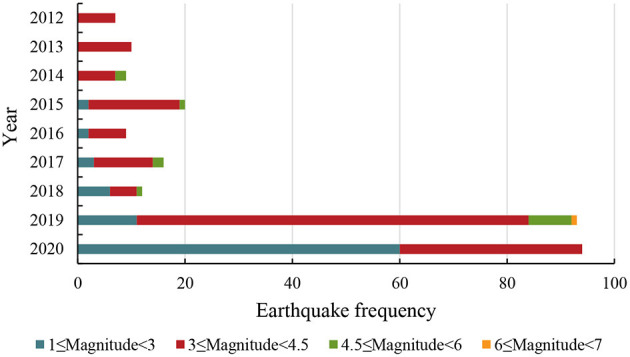
Earthquake frequency and magnitude in Changning County, China and its surrounding areas from 2012 to 2020.

The study site was Changning County, Yibin city, Sichuan Province. The absolute geographic coordinates of Changning County are between 28°15′18″ and 28°47′48″ north latitude and between 104°44′22″ and 105°03′30″ east longitude (Figure 1). The study site was selected for two reasons. The first reason is that a destructive earthquake occurred in this area. The second reason is that the geological movement in this area has been active in recent years, and earthquakes have occurred frequently (34). In the past 8 years, ~270 earthquakes have occurred in Changning County and its surrounding areas. Among these earthquakes, nearly 94 occurred in 2020 (Figure 2). Thus, it is a typical area with frequent earthquakes. According to the presurvey we conducted, local rural residents believed that shale gas extraction in this region has caused frequent earthquakes in recent years, so they have attached great importance to housing safety. This opinion was conducive to the survey of this study. In 2019, the per capita disposable income of rural residents in Changning County was CNY 17,470, which was categorized as neither poor nor developed in China. Earthquakes notwithstanding, the county is a relatively normal place.

**Figure 3:**
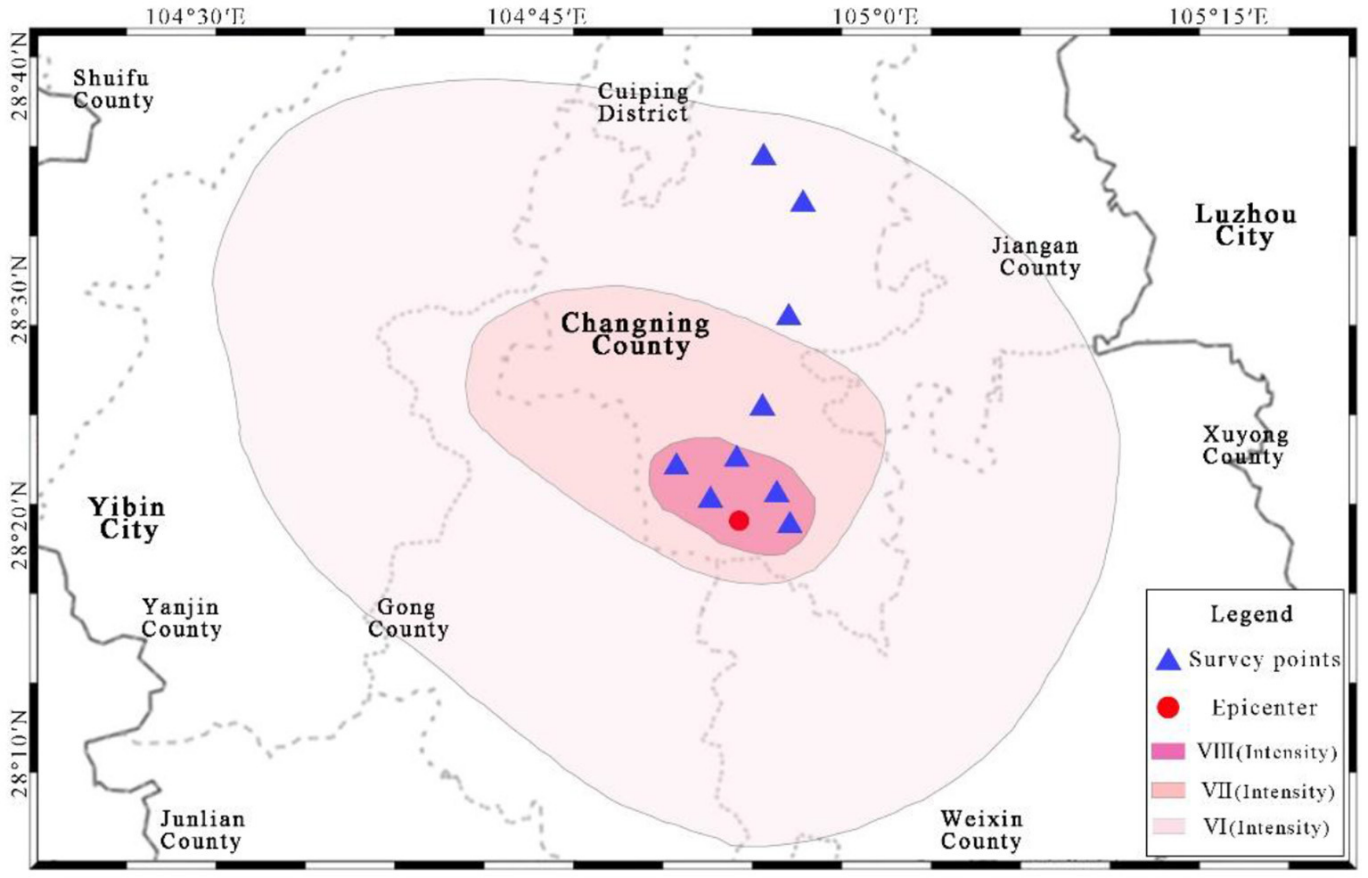
Distribution of the survey points and the earthquake intensity of the 2019 6.0-magnitude earthquake in Changning County, China (adapted from the China Earthquake Administration, 2019).

Nine villages were randomly selected near the epicenter of the 6.0-magnitude earthquake in Changning County, China in 2019 (Figure 3). In this area, the houses of rural residents are in large-scale scattered and small-scale concentrated states. We attempted to conduct one-to-one interviews with all rural residents at home in the investigated settlements, and we completed the questionnaires based on oral statements. To accurately judge and evaluate the factors of housing safety and structural forms, six master's in architecture students conducted the field investigation. Before the investigation, they were educated in mental health analysis and housing safety state assessment skills to ensure the consistency of the questionnaire. Ultimately, 182 questionnaires were sent out, and 162 questionnaires were valid. This field investigation was performed in December 2019.

### Questionnaire Design

The questionnaire was designed to include two sets of questions. One was concerned with the Kessler 6 mental health scale, and the other focused on the living intentions of rural residents and the degree of housing safety (Appendix 1). The main reason for this design is to provide a data foundation for analyzing, through SPSS statistical software, regarding whether the degree of housing safety in seismically active regions can affect the mental health of rural residents, thereby exploring the relationship between housing safety and the mental health of rural residents and developing a new approach for applying telemedicine to assess and prioritize the mental health of rural residents quickly and effectively.

Screening scales are typically used in a multigating assessment framework to identify respondents for subsequent evaluation rather than as diagnostic tools in and of themselves. As such, effective screening scales need to be brief, self-administered, easy to score, comprehensive in their measurement of a range of psychological problems, and clinically relevant (35). The Kessler 10 and 6 scales were originally developed to screen for non-specific psychological distress in serious mental health research. Their reliability and validity have been demonstrated in studies worldwide (36, 37). We chose the Kessler 6 scale for two reasons. First, Kessler 10 and 6 were proved to have almost the same reliability (36, 37). Second, during the interviews, using Kessler 6 can improve the validity of the questionnaire because it is shorter and more convenient for communication and accurate expression of questions. Thus, we used the Chinese version of the K6 questionnaire, which has been validated and is thus reliable (38–40).

The K6 questionnaire was divided into six questions that required participants to answer the frequency they experienced each of six symptoms of mental problems in the month before the questionnaire interview utilizing the response options “never,” “rarely,” “some,” “most,” and “all.” These questions included the frequency of depression; nothing can make them happy, nervous, or upset; despair; everything is an effort; and no value. The score range of the answers was 0–4, and a scale of 0–24 was generated. The higher the score was, the worse the interviewee's mental health. Scores of 0–12 points were regarded as having no mental problems, and scores of 13–24 points were regarded as having mental problems.

The living intentions of rural residents included two questions. The first was the place where residents wanted to live most, and the second was whether rural residents have considered the influence of earthquakes. This question is the premise and foundation to study the relationship between the degree of housing safety and the mental health of rural residents. It can analyze the importance of housing to rural residents even though they are facing frequent earthquakes and then prove the necessity of this study, as well as the effectiveness and rationality of degree of housing safety factor design. The places where residents most want to live were divided into 5 categories, namely, current housing without renovation, renovated current housing, new rural community, suburban district and urban district. The classification was based on the regional categories in China's urban and rural planning.

The selection of the evaluation factors of the degree of rural housing safety is an important part of the questionnaire design. First, the satisfaction of rural residents with the degree of safety of their housing was included in the study. This factor is the foundation of this study. The interviewers with architectural backgrounds also scored the degree of housing safety, which can improve the validity of the questionnaire combined with the scores of other questions. Finally, many existing studies evaluated the state of housing safety using factors such as the building type, structural forms, degree of wall tilt, and degree of wall cracks (41). These objective factors have been widely applied to housing safety assessments. Therefore, based on the characteristics of the research object, the following questions were designed from subjective and objective aspects:

(1) The satisfaction of rural residents with the degree of safety of their housing was scored in the range of 1–5, in which 1 was “very dissatisfied” and 5 was “very satisfied.”
(2) The degree of housing safety scored by interviewers was scored in the range 1–5, in which 1 was “very bad” and 5 was “very good.”
(3) The housing structure forms were divided into four types. The first group was the masonry housing, and the judgment was based on the housing using the masonry walls as the load-bearing structure. The second group was brick-concrete housing, and the judgment was based on the housing using masonry walls as the main load-bearing structure and using reinforced concrete to assist the masonry walls. The third group was wooden structure housing, and the judgment was based on the housing using wood as the load-bearing structure. The fourth group was reinforced concrete frame structure housing, and the judgment was based on the housing using a frame comprised of columns and using reinforced concrete as the load-bearing structure of the house.
(4) The degree of wall tilt was divided into four levels. Level 1 was “not at all,” level 2 was “not obvious,” level 3 was “general,” level 4 was “obvious,” and level 5 was “very obvious.”
(5) The degree of wall cracks was divided into four levels. Level 1 was “not at all,” level 2 was “not obvious,” level 3 was “general,” level 4 was “obvious,” and level 5 was “very obvious.”

### Data Analysis

SPSS statistical software was used to analyze the collected data from the questionnaire survey. First, the living intentions of rural residents were analyzed using descriptive statistics. Next, unitary linear regression analysis and binary logistics regression analysis were conducted to assess the satisfaction of rural residents with the degree of safety of their housing and K6 score and whether rural residents suffer from mental problems, respectively. Third, one-way ANOVA was conducted for the four groups of different housing structure forms and K6 scores, and the chi-squared test was also conducted to determine the association between whether rural residents live in a reinforced concrete frame house and whether they suffer from mental problems. Finally, the degree of housing safety scored by interviewers, the degree of wall tilt, and the degree of wall cracks as independent variables were analyzed by a multiple linear regression to test whether they directly affected the K6 score. Finally, logistic regression analysis was adopted to explore the relationship between the degree of wall tilt and the degree of wall cracks and whether rural residents suffer from mental problems.

## Results

### Living Intention of Rural Residents

**Figure 4:**
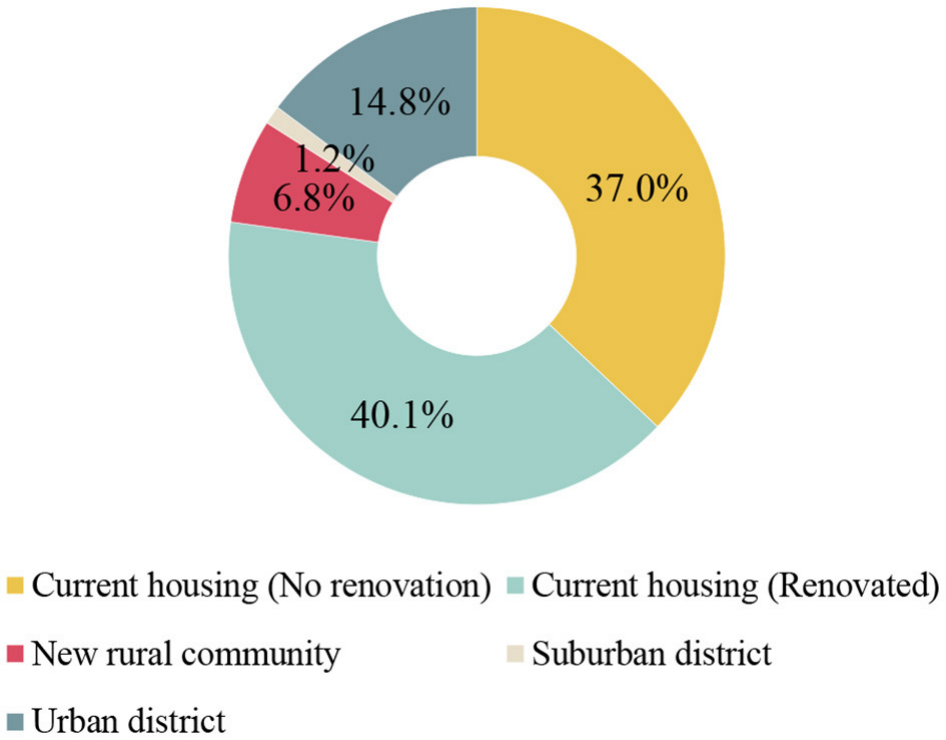
Percentages for the places where rural residents want to live the most.

Figure 4 shows the percentages of the places where rural residents want to live the most. The most desirable place for rural residents to live was their current housing that has been renovated, followed by their current housing that has not been renovated (37.0 and 40.1%, respectively). The percentage for living in a suburban district was the smallest, and only a minority of the population wanted to live in a new rural community or an urban district. Furthermore, 72.2% of rural residents said they made their living intention choice after considering the influence of earthquakes.

### Regression Analysis of Satisfaction With Housing Safety, K6 Score, and Mental Problems

Unitary linear regression and logistic regression analysis showed significant correlations between housing safety satisfaction and the K6 score and whether rural residents suffer from mental problems (*p* = 0.000 and *p* = 0.000, respectively) (Table 1). High satisfaction with housing safety was a protective factor for mental problems (OR = 0.454).

**Table 1:** Regression analysis of satisfaction with housing safety, K6 score, and mental problems.

	**K6 score**			**Whether rural residents suffer from mental problems**		
	***p***	**Unstandardized coefficients**	**t**	**OR**	**95% CI**	***p***
Satisfaction of housing safety	0.000	13.001	11.017	0.454	0.321–0.643	0.000

### Relationship Between Housing Structural Forms, K6 Score, and Mental Problems

Tables 2–4 show significant differences in K6 scores between groups, indicating that the mental health of rural residents with different structural forms was significantly different. The highest mean value and standard deviation of the K6 score were for wooden structure housing (mean value = 9.17, standard deviation = 5.718), and the lowest were for reinforced concrete frame structure housing (mean value = 3.73, standard deviation = 4.245). Therefore, the rural residents with the best mental state were those who lived in reinforced concrete frame structure housing whereas those with the worst mental state was those who lived in wooden structure housing. In addition, the K6 score of reinforced concrete frame structure housing was significantly different from those of brick-concrete housing (sig = 0.001) and wooden structure housing (sig = 0.002), while no significant difference was found between the other groups.

**Table 2:** Descriptive analyses of K6 score.

	***N***	**Mean**	**Std. deviation**	**Std. error**	**95% CI for mean**	**Minimum**	**Maximum**
Masonry housing	18	6.22	4.427	1.043	4.02–8.42	0	17
Brick-concrete housing	110	7.56	5.031	0.480	6.61–8.51	0	21
Wooden structure housing	12	9.17	5.718	1.651	5.53–12.80	2	16
Reinforced concrete frame structure housing	22	3.73	4.245	0.905	1.85–5.61	1	17
Total	162	7.01	5.088	0.400	6.22–7.80	0	21

**Table 3:** Results of one-way ANOVA.

	**Sum of squares**	**df**	**Mean square**	***F***	**Sig**.
Between groups	337.779	3	112.593	4.645	0.004
Within groups	3,830.196	158	24.242		
Total	4,167.975	161			

*df, degrees of freedom; F, the ratio of the mean square between groups to the mean square within groups*.

**Table 4:** Multiple comparisons between groups.

**(I) Influence intensity of housing structural forms**	**(J) Influence intensity of housing structural forms**	**Mean difference (I–J)**	**Std. error**	**Sig**.	**95% CI**
Masonry housing	Brick-concrete housing	−1.341	1.252	0.286	−3.81–1.13
	Wooden structure housing	−2.944	1.835	0.111	−6.57–0.68
	Reinforced concrete frame structure housing	2.495	1.565	0.113	−0.60–5.59
Brick-concrete housing	Wooden structure housing	−1.603	1.497	0.286	−4.56–1.35
	Reinforced concrete frame structure housing	3.836^*^	1.150	0.001	1.57–6.11
Wooden structure housing	Reinforced concrete frame structure housing	5.439^*^	1.767	0.002	1.95–8.93

* *Indicates that the mean difference is significant at the 0.05 level*.

The results shown in Tables 5, 6 shows no significant relationship between the prevalence of mental problems of residents who have moved into reinforced concrete frame structure housing and those who have not (sig = 0.114).

**Table 5:** Cross-tabulation of the groups and description of mental problems.

			**Description of mental problems**		**Total**
			**Absence of mental problems**	**Presence of mental problems**	
Groups	People who do not live in reinforced concrete frame structures housing	Number	110	30	140
		Percentage	78.6%	21.4%	100.0%
	People who live in reinforced concrete frame structures housing	Number	21	1	22
		Percentage	95.5%	4.5%	100.0%

**Table 6:** Chi-squared test: housing structure forms affecting mental problems.

	**Value**	**df**	**Asymp. sig**.	**Exact sig**.	**Exact sig**.
Pearson on Chi-squared	3.502^a^	1	0.061		
Continuous correction^b^	2.496	1	0.114		
Likelihood ratio (L)	4.554	1	0.033		
Fisher exact test				0.079	0.046
Linear-by-linear association	3.481	1	0.062		
N of valid cases	162				

a *0 cells (25.0%) have expected count less than 5. The minimum expected count is 4.21*.

b *Computed only for a 2 × 2 table*.

### Housing Safety Factors Affecting the K6 Score and Mental Problems

Table 7 shows the Pearson correlation analysis. The table shows the statistical significance of the degree of housing safety scored by interviewers (−0.366^**^), the degree of wall tilt (0.365^**^), and the degree of wall cracks (0.458^**^). Strong correlations were found between other factors except the dependent variable, suggesting that multiple linear regression analysis can be adopted. Therefore, we used multiple linear regression analysis, and the results are given in Table 8. First, no significant linear relationship was found between the degree of housing safety scored by interviewers, the degree of wall tilt, and the K6 score (sig = 0.058 and sig = 0.595, respectively). Finally, the degree of wall cracks significantly affected the mental health of rural residents (sig = 0.000).

**Table 7:** Correlation analysis of safety factors and K6 score.

	**Degree of housing safety scored by interviewers**	**Degree of wall tilt**	**Degree of wall cracks**
K6 score	−0.366^**^	0.365^**^	0.458^**^
Degree of housing safety scored by interviewers	1	−0.572^**^	−0.500^**^
Degree of wall tilt	−0.572^**^	1	0.639^**^
Degree of wall cracks	−0.500^**^	0.639^**^	1

*Pearson correlation coefficients*.

** *p < 0.01*.

**Table 8:** Coefficients of Model 1.

**Model**		**Unstandardized coefficients**		**Standardized coefficients**	***t***	**Sig**.	**Collinearity statistics**	
		**B**	**Std. error**	**Beta**			**Tolerance**	**VIF**
1	(Constants)	6.372	2.049		3.111	0.002		
	Degree of housing safety scored by interviewers	−0.800	0.419	−0.166	−1.908	0.058	0.642	1.557
	Degree of wall tilt	0.261	0.491	0.052	0.532	0.595	0.507	1.973
	Degree of wall cracks	1.317	0.357	0.342	3.692	0.000	0.565	1.770

*The dependent variable is the K6 score*.

Table 9 shows that among the two independent variables included, the degree of wall tilt (sig. = 0.117, 95% CI = 0.909–2.360) had no statistical relationship with whether rural residents suffered from mental problems. However, the degree of wall cracks significantly affected the mental health of rural residents (sig. = 0.000, 95% CI = 1.438–3.330), and this factor was a risk factor for mental health (OR = 2.118).

**Table 9:** Logistic regression analysis of potential building safety factors for mental problems.

		**B**	**S. E**	**Wald**	**Sig**.	**OR**	**95% CI**
Step 1^a^	Degree of wall tilt	0.381	0.243	2.453	0.117	1.464	0.909–2.360
	Degree of wall cracks	0.783	0.214	13.361	0.000	2.188	1.438–3.330
	(Constant)	−4.290	0.620	47.798	0.000	0.014	

a *Variable(s) entered on step 1: degree of wall tilt, degree of wall cracks*.

## Discussion

In this paper, we studied the relationship between the degree of housing safety and the mental health of rural residents in seismically active regions. The results show that the higher the degree of housing safety is, the better the mental status of rural residents, and vice versa. To the best of our knowledge, very few studies have assessed the relationship between housing safety and the mental health of rural residents in seismically active regions in Southwest China. This study is relatively representative in the interdisciplinary field of architecture and public health. These findings enable us to propose suggestions for improving housing safety to foster the mental health of local rural residents and develop a new method to assess and prioritize the mental health of rural residents by using degrees of housing safety and smart technology in seismically active regions.

### Relationship Between Housing Safety Degree and Mental Health

First, even with frequent earthquakes, most rural residents preferred to live in their own housing. Therefore, the degree of housing safety in seismically active regions may be one of the greatest factors affecting the mental health of Chinese rural residents. This provides a foundation for the need for housing improvement and the telemedicine assessment method proposed in the following paper.

Second, the satisfaction of rural residents with the degree of safety of their housing significantly affected the mental health of rural residents, which also corresponds to other studies. The degree of housing destruction after an earthquake is a risk factor affecting the mental health of residents, as has been shown in previous studies (6, 7). This provides a meaningful reference for rural disaster prevention in seismically active regions, that is, actively improving housing safety. In addition, further studying and improving the factors affecting health, including psychological and environmental aspects, is necessary to achieve the continuous improvement of public health and maintain social fairness and justice in these areas.

**Figure 5:**
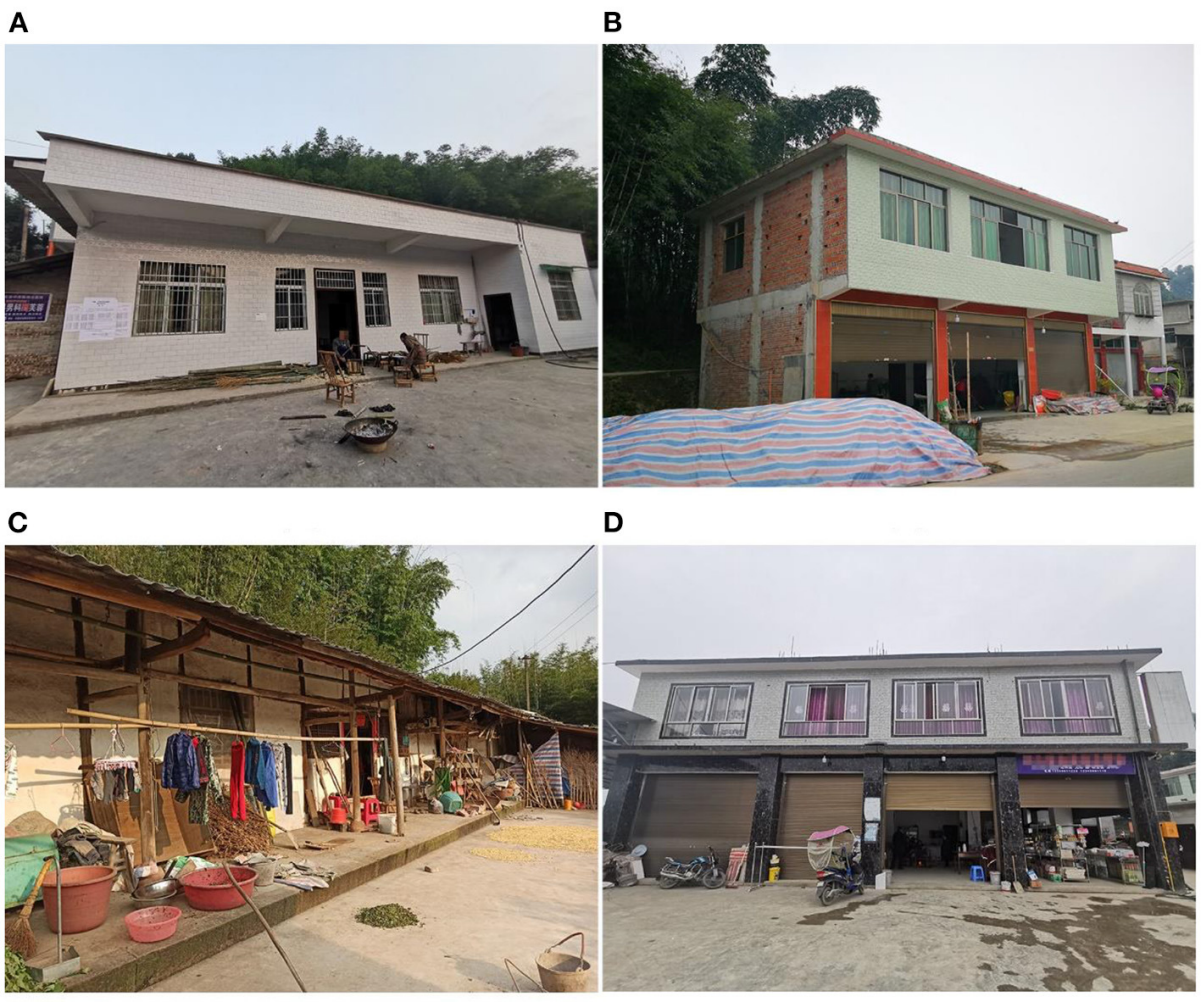
Four types of housing structure forms: **(A)** Masonry housing, **(B)** Brick-concrete housing, **(C)** Wooden structure housing, and **(D)** Reinforced concrete frame structure housing.

Third, the mental health of rural residents who live in reinforced concrete frame structure housing was obviously superior to that of those living in brick-concrete housing and wood structure housing. However, no significant difference was found with the mental health of those living in masonry housing (Figure 5). Our analysis found that most of the masonry housing in the survey comprised one-story buildings. Their construction age was relatively new, and they performed well in earthquakes. Thus, residents were satisfied with their living conditions. The brick-concrete structure was different from the wooden structure. Multistory brick concrete housing had the worst seismic performance and the most collapses. Thus, its performance not being as good as that of the frame structure was expected. Existing research shows that the seismic performance of traditional Chinese wooden buildings is excellent (42). However, the mental health K6 scores of its residents were very poor. Our summary of the interview content shows that the wooden structure houses investigated were old, the oldest of which was nearly 100 years old. These wooden structures had poor living conditions. Previous studies also showed that housing quality significantly influenced mental health status (43). Reinforced concrete frame structure housing itself has good seismic performance, crack resistance, and durability. These structures received residents' recognition. Thus, reinforced concrete frame structure housing is a better choice but has the disadvantage of having relatively high costs. Overall, a reinforced concrete frame structure is preferred. If the budget is insufficient, one-story masonry housing with reinforcement measures can be considered.

**Figure 6:**
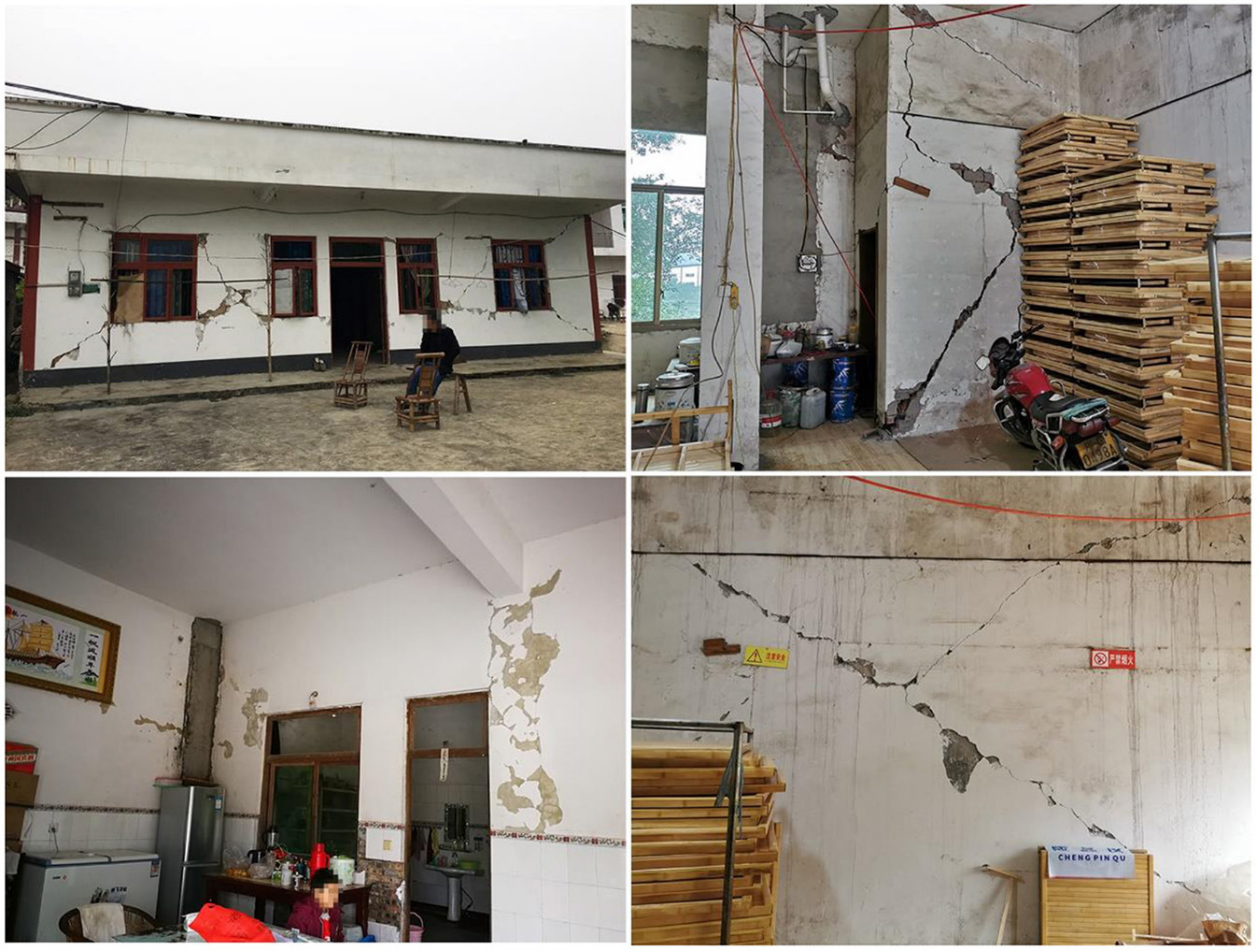
State of wall cracks in the houses of rural residents.

In addition, the core finding of the study is that although wall cracks have little effect on the degree of housing safety, rural residents may subconsciously think that the house is unsafe when they see cracks, prompting dissatisfaction, depression and mental health problems (Figure 6). Thus, if the government needs to improve the mental state of rural residents but is unable to cover all the costs for housing safety improvement, an effective approach is to repair the wall cracks in rural residents' houses. For example, using improved crack-resistance plastering or coating to improve and repair the cracks in the walls after an earthquake can improve the perceived degree of housing safety.

### Telemedicine Assessment for Mental Health Based on Housing Safety Degree

**Figure 7:**
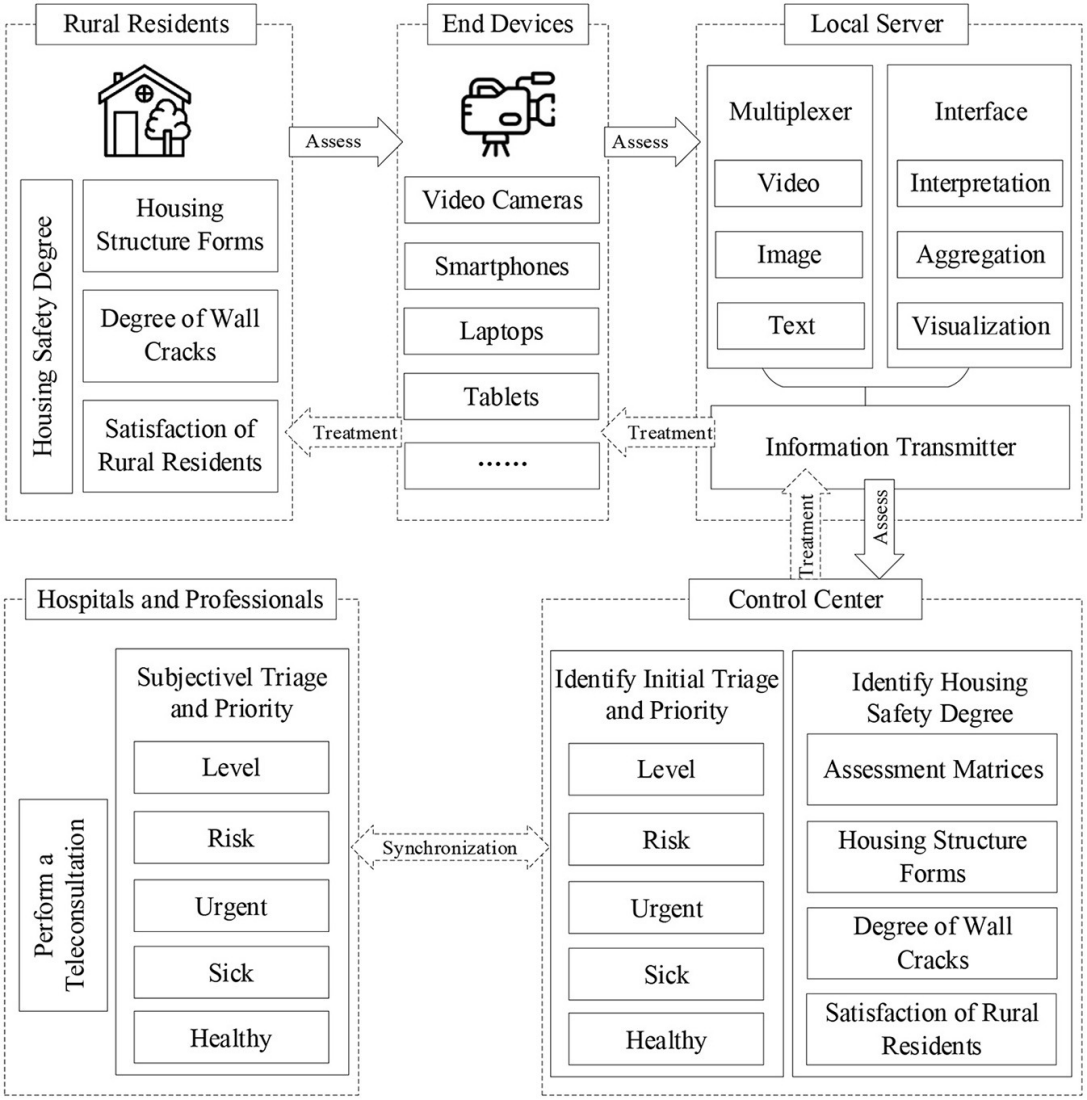
Design of telemedicine assessment architecture for mental health based on housing safety degree.

Based on the correlation between housing safety degree and mental health of rural residents described above, we present a new approach to assess and prioritize the mental health of rural residents by using the degree of housing safety and smart technology in seismically active regions (Figure 7). The telemedicine assessment approach is expected to be used for the mental health triage and large-scale data scoring of rural residents.

As shown in Figure 7, the system consists of (1) the following end devices: video cameras, smartphones, laptops, tablets, video kiosks; (2) local server that collects the information of housing from these end devices such as digital videos and images captured by video cameras; (3) a control center that is an integrated computer located in hospitals or mental health care providers, and dynamically processes the data and assist psychologists and nurses make a triage. Next, the implement steps and each component of the telemedicine assessment was designed as follows:

(1) The digital videos and images of rural residents' housing in seismically active regions can be recorded by local volunteers and government workers, and then these recordings are multiplexed, transformed and transmitted to the control center by the local sever.
(2) The control center deployed in remote hospitals communicates with all the corresponding local severs. Upon successfully receiving the information of housing, the control center identifies the information, utilizes a smart algorithm to make an initial assessment of the degree of housing safety and detects the highest and lowest priority levels, which will make assessment time shorter and enable reaching the most affected rural residents better.
(3) Furthermore, for subjective validation, psychologists and nurses can use data from the control center to confirm the ranking results and achieve final triage. Next, according to the results of the initial assessment and professional evaluation, the control center divides these rural residents into several groups.
(4) Rural residents with potential and risky mental problems will be informed to perform a teleconsultation with remote psychologists by the local server that receives transmitted information from the control center.
(5) Finally, remote psychologists will conduct teleconsultation and treatment using the end devices with live interactive functions, such as via web-based cameras on smartphones, laptops, tablets, or video kiosks.

Our present study had some strengths. First, this study is one of the few studies using data related to seismically active regions, with a particular focus on the degree of housing safety in the area (6, 7, 11). Our research also has certain limitations. The results in this study reflected data from a county in a seismically active region in China. A survey conducted in a larger area may increase the accuracy of the results. However, this study is a preliminary exploration. The research sample is representative and can effectively describe the relationship between the degree of housing safety and the mental health of rural residents. In future research, we will continue to focus on the mental health of rural residents.

## Conclusion and Future Work

In this paper, we presented a new telemedicine approach for assessing and prioritizing the mental health of rural residents in seismically active regions in Southwest China based on the degree of housing safety. The highlights regarding how our work advances the field from the present state of knowledge are as follows:

(1) In seismically active regions in Southwest China, the degree of housing safety could affect the mental health of rural residents. The higher the degree of housing safety is, the better the mental state of rural residents; conversely, the worse their mental health.
(2) Rural residents' satisfaction with the degree of housing safety significantly affected their mental state.
(3) The degree of wall cracks was the largest risk factor for the mental health of the residents. From the perspective of architecture, the development of anticrack coating is the primary measure to improve the mental health of rural residents. Moreover, the factor can be used for the foundation of telemedicine assessment because it is easy to extract and judge.
(4) A new approach was developed to assess and prioritize the mental health of rural residents by using degrees of housing safety and smart technology in seismically active regions. The telemedicine assessment approach is expected to be used for mental health evaluation and large-scale data scoring of rural residents.

Future work could include a survey conducted in a larger area, enrichment of the psychological factors of rural residents and the software development of a smart telemedicine assessment tool. This would help in the treatment of the mental problems of rural residents with more detailed functional factors and would also allow the automatic assessment and prioritization of mental health. The degree of housing safety is not the only factor that affects the mental health of rural residents in seismically active regions. Improving housing safety is not the only way to improve the mental state of rural residents. However, these improvements and the implementation of the concept of telemedicine in seismically active regions have positive effects on rural residents mentally and materially. The entire telemedicine assessment-based degree of housing safety of rural residents is not a trivial task, requires the cooperation of several stakeholders and is part of our future plan.

## Data Availability Statement

The original contributions presented in the study are included in the article/supplementary material, further inquiries can be directed to the corresponding author/s.

## Ethics Statement

The studies involving human participants were reviewed and approved by Chongqing University Committee.

## Author Contributions

YP and RX drafted the manuscript, revised it critically, and contributed substantially to the conception of the project as well as to the analysis and the interpretation of data. QY revised the manuscript critically, commented extensively on it during the review, and revision. TZ commented extensively on the manuscript. All authors have read and approved the submitted manuscript.

## Conflict of Interest

The authors declare that the research was conducted in the absence of any commercial or financial relationships that could be construed as a potential conflict of interest.

## Publisher's Note

All claims expressed in this article are solely those of the authors and do not necessarily represent those of their affiliated organizations, or those of the publisher, the editors and the reviewers. Any product that may be evaluated in this article, or claim that may be made by its manufacturer, is not guaranteed or endorsed by the publisher.

## Appendix

**Appendix 1:** Content of questionnaire.

Kessler 6 mental health scale				
1. Do you feel nervous for no apparent reason?				
(1) Never	(2) Rarely	(3) Some	(4) Most	(5) all
2. Do you feel that your life is in despair?				
(1) Never	(2) Rarely	(3) Some	(4) Most	(5) all
3. Do you feel unrested and insecure?				
(1) Never	(2) Rarely	(3) Some	(4) Most	(5) all
4. Do you feel like there is nothing to cheer you up?				
(1) Never	(2) Rarely	(3) Some	(4) Most	(5) all
5. Did you feel everything requires effort?				
(1) Never	(2) Rarely	(3) Some	(4) Most	(5) all
6. Have you been feeling worthless for the last month or so?				
(1) Never	(2) Rarely	(3) Some	(4) Most	(5) all
Degree of housing safety and the living intentions of rural residents				
1. Are you satisfied with the degree of safety of your housing?				
(1) Very dissatisfied	(2) Less satisfied	(3) General	(4) Satisfied	(5) Very satisfied
2. The degree of housing safety scored by interviewers:				
(1) Very bad	(2) Bad	(3) General	(4) Good	(5) Very good
3. The housing structure forms:				
(1) Masonry housing	(2) Brick-concrete housing	(3) Wooden structure housing	(4) Reinforced concrete frame structure housing	
4. The degree of wall tilt:				
(1) Not at all	(2) Not obvious	(3) General	(4) Obvious	(5)Very obvious
5. The degree of wall cracks:				
(1) Not at all	(2) Not obvious	(3) General	(4) Obvious	(5)Very obvious
6. If conditions allow, where would you most like to live? Have you considered the influences of earthquakes?				
(1) Current housing without renovation	(2) Renovated current housing	(3) New rural community	(4) Suburban district	(5) Urban district
